# Characterization of microRNA expression in bovine adipose tissues: a potential regulatory mechanism of subcutaneous adipose tissue development

**DOI:** 10.1186/1471-2199-11-29

**Published:** 2010-04-27

**Authors:** Weiwu Jin, Michael V Dodson, Stephen S Moore, John A Basarab, Le Luo Guan

**Affiliations:** 1 Department of Agricultural, Food and Nutritional Science, University of Alberta, Edmonton, AB, T6G2P5, Canada; 2 Department of Animal Sciences, Washington State University, PO Box 646310, Pullman, Washington, 99164 USA; 3 Alberta Agriculture and Rural Development, Lacombe Research Centre, Lacombe, AB, T4L1W1, Canada

## Abstract

**Background:**

MicroRNAs (miRNAs), a family of small non-coding RNA molecules, appear to regulate animal lipid metabolism and preadipocyte conversion to form lipid-assimilating adipocytes (*i.e. *adipogenesis). However, no miRNA to date has been reported to modulate adipogenesis and lipid deposition in beef cattle.

**Results:**

The expression patterns of 89 miRNAs including four bovine specific miRNAs in subcutaneous adipose tissues from three groups of crossbred steers differing in backfat thickness were compared using qRT-PCR analysis. Eighty-six miRNAs were detectable in all samples, with 42 miRNAs differing among crossbreds (P < 0.05) and 15 miRNAs differentially expressed between tissues with high and low backfat thickness (P < 0.05). The expression levels of 18 miRNAs were correlated with backfat thickness (P < 0.05). The miRNA most differentially expressed and the most strongly associated with backfat thickness was miR-378, with a 1.99-fold increase in high backfat thickness tissues (r = 0.72).

**Conclusions:**

MiRNA expression patterns differed significantly in response to host genetic components. Approximately 20% of the miRNAs in this study were identified as being correlated with backfat thickness. This result suggests that miRNAs may play a regulatory role in white adipose tissue development in beef animals.

## Background

MicroRNAs (miRNAs) are small regulatory molecules (18~25 nucleotides) that are important in many biological processes including development, differentiation, apoptosis, and metabolism [1-5]. The role of miRNAs in lipid metabolism was first reported in Drosophila, where the deletion of miR-14 increased the levels of triacylglycerol and diacylglycerol [6]. Thereafter, several miRNAs were shown to promote preadipocyte differentiation in the human and mouse [7-9]. Analysis of the 3' UTR from 395 ESTs expressed in 3T3-L1 preadipocytes during conversion into lipid-assimilating adipocytes showed that >70% of the differentially expressed genes may be potentially regulated by miRNAs [10]. A recent study on the expression of 155 miRNAs in human omental and subcutaneous adipose tissues found that the expression of miRNAs was adipose depot specific and that some miRNAs were correlated with the morphology of adipose tissue and adipocyte size [11]. Although the roles of some miRNAs in lipid metabolism [6] and adipocyte formation [7-10] have been demonstrated, no miRNA has been reported to regulate lipid metabolism in beef cattle. Lipid deposition, especially in subcutaneous adipose tissues, is directly associated with the yield and the quality of meat [12]. We hypothesized that microRNAs play a role in the regulation of adipogenesis in bovine adipose tissue and the variation of expression of miRNAs is associated with differences in backfat thickness. To gain insight into the association between subcutaneous adipose tissue thickness and miRNA expression, the expression profiles of 89 miRNAs were determined in beef cattle subcutaneous adipose tissues of crossbred steers with different backfat thickness.

## Results and Discussion

The aim of this study was to identify miRNAs which may play regulatory roles in adipose tissue development in beef cattle.

### The expression of miRNAs differed significantly in response to host genetic components

**Figure 1:**
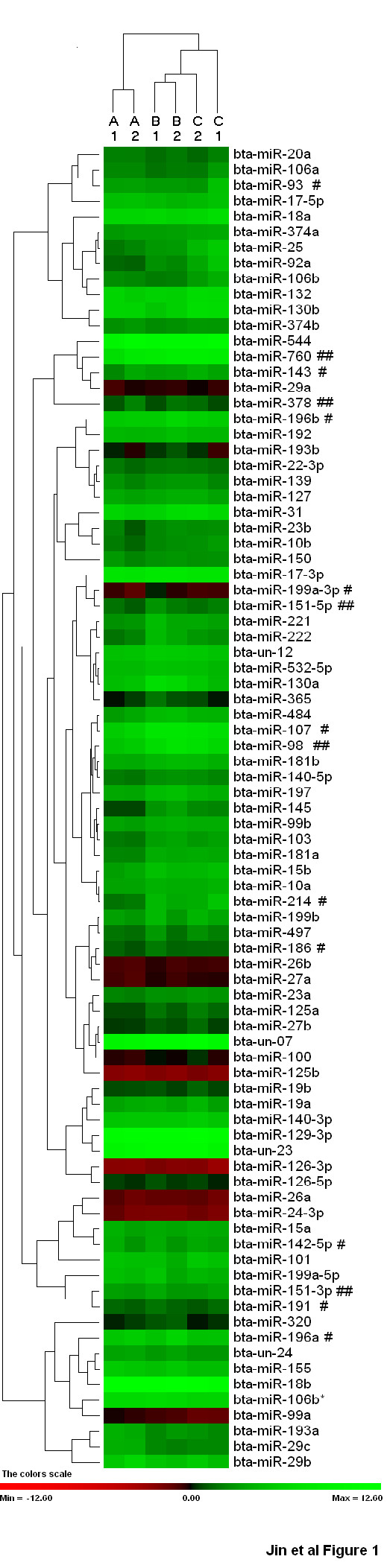
**Expression pattern of miRNAs in back subcutaneous adipose tissues**. Heatmap is constructed based on the mean expression levels of miRNAs in each group (n = 4). As shown by the scale bar, increasing green and red signal intensities indicate miRNA with lower or higher expression level, respectively. A1, CHAM with high backfat; A2, CHAM with low backfat; B1, CHAR with high backfat; B2, CHAR with low backfat; C1, HEAN with high backfat; C2, HEAN with low backfat. '#' and '##' indicate that miRNA expression was significant or very significant difference between tissues with high and low backfat thickness, respectively.

**Figure 2:**
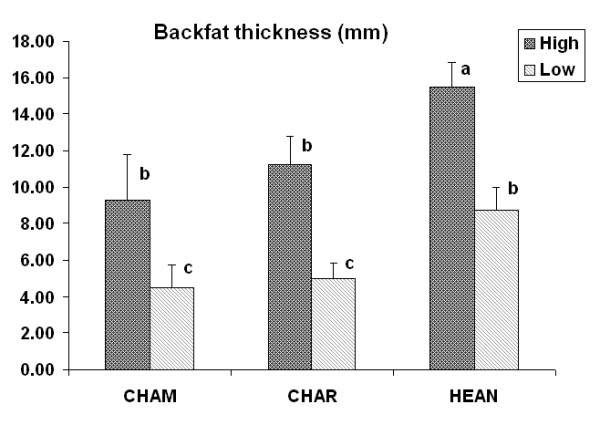
**Comparisons of backfat thickness between groups**. One-way ANOVA was performed. Groups with the same letter are not significantly different.

Analysis of the expression of 89 miRNAs including four bovine specific miRNAs (Additional file 1) in beef cattle back subcutaneous adipose tissues confirmed the expression of 86 miRNAs. The animals were divided into 6 groups, according to breed and level of backfat thickness. Hierarchical clustering of the detectable miRNAs showed the expression of miRNAs was more similar in animals of the same breed composition than animals with the same level of backfat thickness (Figure 1). We also compared miRNA expression among three crossbred groups and found the expression of 42 miRNAs to be significantly different among these groups. These results suggest that the expression of miRNAs may differ with regard to host genetic components. CHAR animals have miRNA expression patterns more similar with those of HEAN animals (Figure 1), with 17 significantly different miRNAs. Twenty-nine and 27 miRNAs were found to be significantly different between CHAM and CHAR crossbreds, and between CHAM and HEAN crossbreds, respectively. However, the backfat thickness of CHAR animals was significantly different than that of HEAN animals and was similar with that of CHAM animals (Figure 2).

### Differentially expressed miRNAs in adipose tissues from animals with different backfat thickness

Fifteen differentially expressed (DE) miRNAs were identified between tissues from high and low backfat thickness (Figure 1, Table 1). Of these 15 DE miRNAs, seven (miR-378, -143, -760, -98, -196a, -196b and -107) were identified as highly expressed in the tissue (> 1.34-fold) from high backfat thickness animals, whereas the remaining eight (miR-93, -151-5p, -214, -151-3p, -199a-3p, -191, -142-5p and -186) were expressed at a higher level in tissue from the low backfat thickness animals (Table 1). The most differentially expressed miRNA was miR-378 with a 1.99-fold increase in high backfat thickness tissues, suggesting that this miRNA may have a role in adipogenesis and/or lipid deposition in bovine backfat tissues.

**Table 1:** Differentially expressed miRNAs between high and low backfat thickness fat tissues.

	High (n = 12)	Low (n = 12)	-Fold change	
bta-miRNA	Mean ± SD	Mean ± SD		p-value
miR-378	0.84 ± 0.38	1.83 ± 0.53	1.99	<0.0001
miR-760	7.95 ± 0.82	8.65 ± 0.43	1.62	0.0005
miR-151-3p	3.51 ± 0.44	3.02 ± 0.15	-1.40	0.0022
miR-151-5p	2.19 ± 0.73	1.59 ± 0.44	-1.52	0.0050
miR-98	6.19 ± 0.95	6.85 ± 1.05	1.58	0.0097
miR-196a	5.10 ± 0.35	5.68 ± 0.81	1.49	0.0101
miR-214	3.79 ± 1.64	3.21 ± 1.03	-1.50	0.0101
miR-93	3.78 ± 1.13	3.08 ± 0.35	-1.62	0.0106
miR-196b	5.56 ± 0.50	6.09 ± 0.53	1.44	0.0148
miR-107	6.83 ± 0.97	7.25 ± 0.81	1.34	0.0173
miR-186	1.55 ± 0.41	1.21 ± 0.34	-1.27	0.0178
miR-199a-3p	-0.29 ± 0.49	-0.69 ± 0.51	-1.32	0.0198
miR-191	1.57 ± 0.46	1.17 ± 0.28	-1.32	0.0202
miR-142-5p	3.69 ± 0.66	3.14 ± 0.57	-1.47	0.0266
miR-143	3.03 ± 0.71	3.76 ± 0.85	1.66	0.0268

N-fold change is calculated with 2^|**Mean**^_**high**_^**-Mean**^_**low**_^|^.

In mouse the miR-17-92 cluster has been reported to promote preadipocyte differentiation [8]; and the miR-17-92 cluster with other two cluster paralogs (miR-106a-363 and miR-106b-25) were found to promote cell proliferation [13]. To test whether the miRNAs of the miR-17-92 family are expressed higher in tissues with high backfat thickness, expression patterns of all members omitting miR-20b (Additional file 1, 2) were profiled. Unexpectedly, no DE miRNA from three clusters were identified, with the exception of miR-93 which was found 1.62-fold down-regulated in tissues from high backfat thickness animals compared to those from low backfat thickness animals (P > 0.05). Our undetected expression difference of the miR-17-92 cluster from this study confirmed the previous observation that bovine adipogenesis had different molecular mechanisms than those in mice based on the study of gene expression patterns [14]. In addition, the miR-17-92 cluster was found to be up-regulated during the early clonal expansion stage of adipogenesis [8] and was not abundantly expressed in differentiated adipocytes [9]. Since backfat tissues are mixture of preadipocytes, adipocytes and other cells, the stage of adipogenesis could be very different from the pure adipocyte cultures. Further study to verify the expression of miR-17-92 using a bovine adipocyte cell line will provide a better understanding of this miRNA cluster in bovine adipogenesis. In addition, miR-143 was 1.66-fold up-regulated in the tissues from high backfat animals, It has been reported that miR-143 was up-regulated during preadipocyte differentiation in human and mouse [7,11,15], suggesting that this miRNA may promote bovine adipogenesis that may accelerate adipose tissue development.

### Backfat thickness associated miRNAs

To confirm the above correlation between miRNA expression levels and backfat thickness, we further investigated the relationship between the expression of miRNAs and backfat thickness using Pearson correlation and simple linear regression analysis. Eighteen miRNAs, representing 20% of all analyzed miRNAs (Table 2), were identified as being significantly associated with backfat thickness in beef animals. Among the backfat thicknessassociated miRNAs, eight were DE miRNAs identified above, including two up-regulated miRNAs (miR-378, -196a) and six down-regulated miRNAs (miR-151-5p, -151-3p, -214, -186, -142-5p and -93) (Table 1). The most differentially expressed miRNA (miR-378) was shown to have the strongest correlation with backfat thickness (r = 0.72). Four bovine specific miRNAs which were identified from our previous study [16] were also confirmed to be expressed in all the analyzed samples. Among them, one miRNA (bta-miR-3432) was significantly correlated to backfat thickness (r = 0.48), suggesting that this bovine specific miRNA may play a role in the adipogenesis of adipose tissues in cattle. The mechanism of how this miRNA influences bovine adipogenesis can be elucidated by studying the expression of this miRNA in bovine adipocyte cells and to identify its target genes.

**Table 2:** Backfat thickness associated miRNAs.

miRNA	Mean ± SD (n = 24)	r (p-value)	miRNA location^1^	Gene description^2^
miR-378	1.33 ± 0.68	0.72 (<0.0001)	Intronic	peroxisome proliferator-activated receptor gamma, coactivator 1 beta (*)
miR-196a	5.39 ± 0.68	0.65 (0.0006)	Intergenic	
bta-miR-3432	3.22 ± 0.47	0.48 (0.0177)	- (Intergenic)	
miR-92a	2.75 ± 1.65	-0.58 (0.0028)	Intronic (Intergenic)	chromosome 13 open reading frame 25 (-)
miR-151-3p	3.27 ± 0.41	-0.53 (0.0076)	Intronic	protein tyrosine kinase 2
miR-151-5p	1.89 ± 0.67	-0.49 (0.0154)	Intronic	protein tyrosine kinase 2
miR-214	3.50 ± 1.37	-0.52 (0.0094)	Intronic	dynamin 3 (similar to dynamin 3)
miR-106b	2.68 ± 0.86	-0.49 (0.0151)	Intronic	mini-chromosome maintenance protein 7
miR-25	3.36 ± 1.53	-0.48 (0.0164)	Intronic	mini-chromosome maintenance protein 7
miR-93	3.43 ± 0.89	-0.43 (0.0343)	Intronic	mini-chromosome maintenance protein 7
miR-199b	3.59 ± 0.86	-0.48 (0.0188)	Intronic	dynamin 1
miR-10b	2.36 ± 0.98	-0.45 (0.0282)	Intergenic	
miR-186	1.38 ± 0.41	-0.44 (0.0319)	Intronic	zinc finger, RAN-binding domain containing 2 (zinc finger protein 265)
miR-23b	2.24 ± 0.84	-0.43 (0.0386)	Intronic (Intergenic)	chromosome 9 open reading frame 3 (-)
miR-132	6.25 ± 0.63	-0.42 (0.0392)	Intergenic	
miR-101	4.82 ± 0.57	-0.42 (0.0412)	Intronic	RNA terminal phosphate cyclase-like
miR-27a	-0.47 ± 0.69	-0.41 (0.0456)	Intergenic	
miR-142-5p	3.41 ± 0.66	-0.41 (0.0487)	Intergenic	

^1^miRNA location is listed. If the human/cattle positions are different, the cattle location is presented in parentheses. Data are from miRBase.

^2^Gene is listed. If the human/cattle genes are different, the cattle gene is presented in parentheses. Data are from miRBase, NCBI and Ensembl.

'-' means not find so far. *This gene in bovine is named IPI00715774.3 (Ensembl) and shares homology to human PGC-1β (Blastp).

Most of backfat thickness associated miRNAs in this study were found to be negatively correlated to backfat depth (Table 2). For example, the miR-106b-25 cluster, a miRNA cluster paralog of miR-17-92, was moderately negatively correlated with backfat thickness. These data suggest that miRNA may regulate white adipose tissue development, and that the difference in thickness of subcutaneous tissues may be one target. More research is needed to verify this and to provide intracellular/gene mechanisms.

### Potential role of miR-378 in bovine adipogenesis

**Figure 3:**
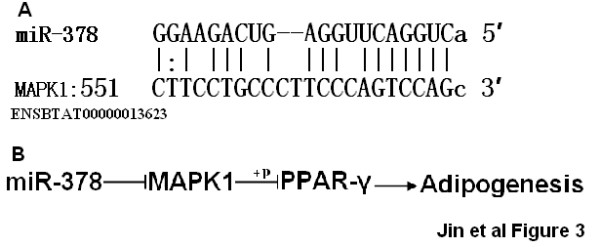
**Hypothesis of miR-378 in bovine adipogenesis**. A. Potential target of miR-378 was predicted in the 3' UTR of MAPK1 (Ensembl ID and position are listed). B. Regulation pathway of miR-378 in adipogenesis.

Approximately two thirds of the miRNAs that were correlated with backfat thickness in this study were identified in introns or exons of genes (Table 2). Intronic miRNAs may be coordinately expressed with their targeted gene mRNAs [17-19] and may act synergistically with the targeted genes [20]. MiR-378, the most DE and the strongest associated with backfat thickness, was found to be located in intron 1 of peroxisome proliferator-activated receptor gamma coactivator 1 beta (PGC-1β) (Table 2). Since PGC-1β has been shown to be induced during brown adipocyte differentiation [21] and to increase lipogenesis and lipoprotein transport in the liver [22,23], we suggest that PGC-1β may play a role in white adipose tissue development. Furthermore, a search of Microcosm Targets V5 http://www.ebi.ac.uk/enright-srv/microcosm/htdocs/targets/v5/ with miR-378 identified 816 potential target genes. One interesting target gene is mitogen-activated protein kinase 1 (MAPK1). MAPK1 can mediate phosphorylation of the dominant adipogenic transcription factor peroxisome proliferator-activated receptor γ (PPARγ) and reduce its transcriptional activity [24-26]. PPARγ is up regulated in bovine preadipocyte adipogenesis, [27,28] and the knock-down of PPARγ markedly inhibited preadipocyte-to adipocyte conversion in 3T3-L1 cells [29]. Therefore, we speculate that miR-378 may promote bovine adipogenesis in white adipose tissue through targeting MAPK1 and PPARγ (Figure 3). However, this regulatory mechanism may be one of the many other functions this miRNA may have. Since miRNA-378 can also target many other genes, future studies on the relationships of miRNA target genes and how the network of these target genes could impact on white adipose tissue development will supply a comprehensive view of the molecular mechanisms of fat formation, a very complicated process in beef cattle.

## Conclusions

We assessed the miRNA expression patterns in the subcutaneous (backfat) adipose depot of crossbred beef cattle and found that the expression patterns of miRNA were different depending on host genetic components. In total, 86 miRNA were detected, and 18 miRNAs were identified as being associated with backfat thickness, suggesting that miRNAs may play an important role in white adipose tissue development in beef cattle. MiRNA-378 was found to be the most DE and strongly associated miRNA with backfat thickness. This miRNA has a potential function of regulating bovine adipogenesis by targeting MAPK1 and of PPARγ. Future work to study miRNA expression of adipose collected from different growth stage, among different adipose tissue depots, or from key individual cell populations derived from adipose tissue proper, is necessary to elucidate the functions of miRNA in bovine lipid metabolism. These will prove useful knowledge to reduce the development of inefficient adipose tissue depots (subcutaneous depot), while enhancing the formation of desired depots (intramuscular depot) in beef cattle. Such knowledge may also apply to humans, especially in terms of regulation of metabolic syndrome, diabetes, or obesity.

## Methods

### Animals and total RNA extraction

All animals in this study were maintained at the Lacombe Research Centre, Lacombe, Alberta, Canada, and were cared for according to the guidelines of the Canadian Council on Animal Care (CCAC 1993). The animal use authorization form was approved by the Animal Care Committee at the Lacombe Research Centre. Hereford × Aberdeen Angus (HEAN) (n = 22), Charolais × Red Angus (CHAR) (n = 13), and Charolais × Maine Anjou (CHAM) (n = 9) crossbred steers were fed on a finishing diet composed of approximately 73.3% barley grain, 22.0% barley silage, 1.6% molasses, and 3.1% feedlot supplement [32% CP beef supplement containing Rumensin (400 mg kg-1) fed *ad libitum*] and slaughtered at 14-17 mo. Details of feeding and management were described by Basarab *et al. *[30]. Carcass traits including backfat thickness, estimated cutability, marbling score, and other information were recorded. The subcutaneous adipose tissues above the 10-11th rib were collected immediately after the animals were harvested at the Lacombe Research Centre abattoir, frozen in liquid nitrogen and stored at -80°C until further analysis. Samples were ranked by backfat thickness at the 12-13th rib (also know as grade fat). Adipose tissues of the top four and the bottom four in each crossbred were selected and divided into high and low backfat thickness groups (Table 3).

**Table 3:** Carcass characteristics of animals used in this study.

Breed	Animal ID	Fat (mm)				Cutability (%)	Age (Day)	Slaughter Weight (kg)
					
		top	mid	bottom	12-13th rib			
CHAR^a^ (Low backfat)	301	8	6	5	4	64	465	571.5
	107	5	5	5	5	64	459	651.5
	403	10	6	6	5	61	488	456.0
	309	10	6	7	6	62	486	557.5
CHAR (High backfat)	106	13	11	11	10	59	458	627.5
	410	15	11	11	10	56	485	504.5
	209	14	12	12	12	55	449	542.5
	210	16	14	14	13	56	476	558.0
CHMA^b^ (Low backfat)	111	6	6	4	3	65	434	578.5
	303	9	5	5	4	62	485	563.5
	407	6	5	5	5	62	476	548.0
	101	6	4	6	6	62	471	687.5
CHMA (High backfat)	201	9	8	7	6	61	467	581.5
	103	10	10	8	8	61	465	634.0
	113	9	9	8	8	61	470	670.5
	112	13	13	13	13	58	462	688.0
HEAN^c^ (Low backfat)	401	13	12	8	7	59	501	540.5
	207	10	9	9	9	58	458	573.5
	205	14	11	10	10	58	463	590.0
	310	13	9	9	9	60	482	576.5
HEAN (High backfat)	405	18	15	14	14	53	494	507.0
	208	19	17	16	15	53	479	519.0
	102	24	19	19	16	54	472	626.5
	203	16	18	18	17	52	459	504.5

^a^CHAR, Charolais × Red Angus; ^b^CHAM, Charolais × Maine Anjou; ^c^HEAN, Hereford × Aberdeen Angus.

Total RNA was extracted with mirVana miRNA Isolation Kit (Ambion Inc, Austin, TX, USA) according to the manufacturer's instruction. The quality and concentration of total RNA were determined by routine agarose gel analysis and spectrophotometer ND-1000 (Thermo Scientific, Waltham, MA, US), respectively.

### MicroRNA expression using qRT-PCR

Expression of 89 miRNAs including four miRNA candidates was carried out with TaqMan^® ^miRNA assays according to the manufacturer's recommendation (Applied Biosystems, Foster City, CA, USA). Briefly, cDNA was reversely transcribed from 10 ng of total RNA using 5× specific miRNA RT primer, respectively. PCR products were amplified from cDNA samples using 20× TaqMan^® ^miRNA Assays. Fluorescence signal was detected on an ABI StepOnePlus Real-time PCR System detector (Applied Biosystems, Foster City, CA, USA). Triplicates or four replicates for each reaction were performed. Bovine miR-16b was used as an endogenous control to normalize the expression levels of the other miRNAs. Eighty-five miRNA assays were selected from mouse, rat or human. Most of them had 100% identity in sequence with bovine miRNAs but some had one or two nucleotides longer or shorter at the 3' or 5' end. Only miR-143 and miR-363 had one and two nucleotides difference at the 3' between bovine and human, respectively (Additional file 1). The other four assays for bovine specific miRNAs (bta-miR-2284w, bta-miR-3431, bta-miR-2284x and bta-miR-3432, Additional file 1) were synthesized by Applied Biosystems (Foster City, CA, USA), and the target sequences were selected from our recently identified bovine miRNA candidates [16].

### Data analyses

MiRNA expression (ΔC_T_) was analyzed by 2-way ANOVA using GLM procedures of SAS 9.2 (2008) for the fixed effects of backfat, crossbred group, backfat × crossbred group. A P value < 0.05 was considered to be statistically significant. miRNA expression between either two crossbred groups was contrasted using 'means breed/Tukey LSD', and the P value was adjusted to 0.017 for statistical significance. The relationships between miRNA expression and backfat thickness were assessed by Pearson's correlation, and simple linear regression was done after the data of backfat thickness were transformed as log2 ratios. The correlation between either two miRNAs was also evaluated by Pearson's correlation. Hierarchical clustering of miRNA expression was performed using PermutMatrix software with Pearson distance for dissimilarity, average linkage for hierarchical clustering and multiple-fragment heuristic for seriation [31].

## Authors' contributions

LLG and WJ designed the experiments; WJ performed qRT-PCR, data analysis, and original manuscript writing; MVD, SSM and JAB were involved in discussion and manuscript construction. WJ and LLG wrote the paper. All authors read and approved the final manuscript.

## Supplementary Material

Additional file 1 TaqMan^® ^miRNA Assays. Click here for file

Additional file 2 The genomic organization of the miR-17-92 family. Click here for file
