# Supplementary material for: Characterization of microRNA expression in bovine adipose tissues: a potential regulatory mechanism of subcutaneous adipose tissue development
Source: BMC Mol Biol. 2010 Apr 27;11:29. doi: 10.1186/1471-2199-11-29 (PMC2874793; doi:10.1186/1471-2199-11-29)
Supplement: Additional file 1 — TaqMan® miRNA Assays. [file 1471-2199-11-29-S1.PDF]

Additional file 1

TaqMan® miRNA Assays

.

Eighty-five miRNA Assays are from mouse, rat or human. Most of them have 100% identity with bovine miRNAs but some have one or two nucleotides longer or shorter at the 3′ or 5′ end (different nucleotide indicated in lowercase). Only miR-143 and miR-363 have one and two nucleotides difference at the 3′ end between bovine and human, respectively (nucleotide variation labeled in red). The other four Assays were synthesized by Applied Biosystems and the target sequences were selected from our recently identified bovine miRNA candidates.

Part #	Assay ID	Assay Name	Target Sequence	Bovine miRNA sequence	miRNA ID
4373153	000387	hsa-miR-10a	UACCCUGUAGAUCCGAAUUUGUG	UACCCUGUAGAUCCGAAUUUGUG	bta-miR-10a
4373152	000388	hsa-miR-10b	UACCCUGUAGAACCGAAUUUGU	UACCCUGUAGAACCGAAUUUGUg	bta-miR-10b
4373123	000389	hsa-miR-15a	UAGCAGCACAUAAUGGUUUGUg	UAGCAGCACAUAAUGGUUUGU	bta-miR-15a
4373122	000390	hsa-miR-15b	UAGCAGCACAUCAUGGUUUACA	UAGCAGCACAUCAUGGUUUACA	bta-miR-15b
4373121	000391	hsa-miR-16	UAGCAGCACGUAAAUAUUGGCG	UAGCAGCACGUAAAUAUUGGCG	bta-miR-16b
4373120	000392	hsa-miR-17-3p	ACUGCAGUGAAGGCACUUGU	ACUGCAGUGAAGGCACUUGU	bta-miR-17-3p
4373119	000393	hsa-miR-17-5p	CAAAGUGCUUACAGUGCAGGUAGU	CAAAGUGCUUACAGUGCAGGUAGU	bta-miR-17-5p
4373118	000394	hsa-miR-18a	UAAGGUGCAUCUAGUGCAGAUA	UAAGGUGCAUCUAGUGCAGAUA	bta-miR-18a
4373184	001009	hsa-miR-18b	UAAGGUGCAUCUAGUGCAGUUA	UAAGGUGCAUCUAGUGCAGUUA	bta-miR-18b
4373099	000395	hsa-miR-19a	UGUGCAAAUCUAUGCAAAACUGA	UGUGCAAAUCUAUGCAAAACUGA	bta-miR-19a
4373098	000396	hsa-miR-19b	UGUGCAAAUCCAUGCAAAACUGA	UGUGCAAAUCCAUGCAAAACUGA	bta-miR-19b
4373286	000580	hsa-miR-20a	UAAAGUGCUUAUAGUGCAGGUAG	UAAAGUGCUUAUAGUGCAGGUAG	bta-miR-20a
4373079	000398	hsa-miR-22	AAGCUGCCAGUUGAAGAACUGU	AAGCUGCCAGUUGAAGAACUGU	bta-miR-22-3p
4373074	000399	hsa-miR-23a	AUCACAUUGCCAGGGAUUUCC	AUCACAUUGCCAGGGAUUUCCa	bta-miR-23a
4373073	000400	hsa-miR-23b	AUCACAUUGCCAGGGAUUACC	AUCACAUUGCCAGGGAUUACCac	bta-miR-23b
4373072	000402	hsa-miR-24	UGGCUCAGUUCAGCAGGAACAG	UGGCUCAGUUCAGCAGGAACAG	bta-miR-24-3p
4373071	000403	hsa-miR-25	CAUUGCACUUGUCUCGGUCUGA	CAUUGCACUUGUCUCGGUCUGA	bta-miR-25
4395166	000405	hsa-miR-26a	UUCAAGUAAUCCAGGAUAGGCU	UUCAAGUAAUCCAGGAUAGGCU	bta-miR-26a
4373069	000406	hsa-miR-26b	UUCAAGUAAUUCAGGAUAGGUU	UUCAAGUAAUUCAGGAUAGGUU	bta-miR-26b
4373287	000408	hsa-miR-27a	UUCACAGUGGCUAAGUUCCGc	UUCACAGUGGCUAAGUUCCG	bta-miR-27a
4373068	000409	hsa-miR-27b	UUCACAGUGGCUAAGUUCUGC	UUCACAGUGGCUAAGUUCUGC	bta-miR-27b
4395223	002112	hsa-miR-29a	UAGCACCAUCUGAAAUCGGUUA	CUAGCACCAUCUGAAAUCGGUUA	bta-miR-29a
4373288	000413	hsa-miR-29b	UAGCACCAUUUGAAAUCAGUGUU	UAGCACCAUUUGAAAUCAGUGUU	bta-miR-29b
4395171	000587	hsa-miR-29c	UAGCACCAUUUGAAAUCGGUUA	UAGCACCAUUUGAAAUCGGUUA	bta-miR-29c

4395390	002279	hsa-miR-31	AGGCAAGAUGCUGGCAUAGCU	AGGCAAGAUGCUGGCAUAGCU	bta-miR-31
4395169	000431	hsa-miR-92a	UAUUGCACUUGUCCCGGCCUGU	UAUUGCACUUGUCCCGGCCUGU	bta-miR-92a
4373302	001090	mmu-miR-93	CAAAGUGCUGUUCGUGCAGGUAg	CAAAGUGCUGUUCGUGCAGGUA	bta-miR-93
4373008	000435	hsa-miR-99a	AACCCGUAGAUCCGAUCUUGUG	AACCCGUAGAUCCGAUCUUGUG	bta-miR-99a
4373007	000436	hsa-miR-99b	CACCCGUAGAACCGACCUUGCG	CACCCGUAGAACCGACCUUGCG	bta-miR-99b
4373160	000437	hsa-miR-100	AACCCGUAGAUCCGAACUUGUG	AACCCGUAGAUCCGAACUUGUG	bta-miR-100
4395364	002253	hsa-miR-101	UACAGUACUGUGAUAACUGAA	UACAGUACUGUGAUAACUGAA	bta-miR-101
4373158	000439	hsa-miR-103	AGCAGCAUUGUACAGGGCUAUGA	AGCAGCAUUGUACAGGGCUAUGA	bta-miR-103
4395280	002169	hsa-miR-106a	AAAAGUGCUUACAGUGCAGGUAg	AAAAGUGCUUACAGUGCAGGUA	bta-miR-106a
4373155	000442	hsa-miR-106b	UAAAGUGCUGACAGUGCAGAU	UAAAGUGCUGACAGUGCAGAU	bta-miR-106b
4395491	002380	hsa-miR-106b*	CCGCACUGUGGGUACUUGCUGC	CCGCACUGUGGGUACUUGCUGC	bta-miR-106b*
4373154	000443	hsa-miR-107	AGCAGCAUUGUACAGGGCUAUCa	AGCAGCAUUGUACAGGGCUAUC	bta-miR-107
4373150	000446	hsa-miR-124a	UUAAGGCACGCGGUGAAUGCCA	UUAAGGCACGCGGUGAAUGCCA	bta-miR-124a
4373149	000448	hsa-miR-125a	UCCCUGAGACCCUUUAACCUGUG	UCCCUGAGACCCUUUAACCUGUG	bta-miR-125a
4373148	000449	hsa-miR-125b	UCCCUGAGACCCUAACUUGUGA	UCCCUGAGACCCUAACUUGUGA	bta-miR-125b
4395339	002228	hsa-miR-126	UCGUACCGUGAGUAAUAAUGCG	CGUACCGUGAGUAAUAAUGCG	bta-miR-126-3p
4373269	000451	hsa-miR-126*	CAUUAUUACUUUUGGUACGCG	CAUUAUUACUUUUGGUACGCG	bta-miR-126-5p
4373147	000452	hsa-miR-127	UCGGAUCCGUCUGAGCUUGGCU	UCGGAUCCGUCUGAGCUUGGCU	bta-miR-127
4373297	001184	mmu-miR-129-3p	AAGCCCUUACCCCAAAAAGCAU	AAGCCCUUACCCCAAAAAGCAU	bta-miR-129-3p
4373171	000590	hsa-miR-129	CUUUUUGCGGUCUGGGCUUGC	CUUUUUGCGGUCUGGGCUUGCU	bta-miR-129-5p
4373145	000454	hsa-miR-130a	CAGUGCAAUGUUAAAAGGGCAU	CAGUGCAAUGUUAAAAGGGCAU	bta-miR-130a
4373144	000456	hsa-miR-130b	CAGUGCAAUGAUGAAAGGGCAU	CAGUGCAAUGAUGAAAGGGCAU	bta-miR-130b
4373143	000457	hsa-miR-132	UAACAGUCUACAGCCAUGGUCG	UAACAGUCUACAGCCAUGGUCG	bta-miR-132
4395400	002289	hsa-miR-139-5p	UCUACAGUGCACGUGUCUCCAG	UCUACAGUGCACGUGUCUCCAGu	bta-miR-139
4395345	002234	hsa-miR-140-3p	UACCACAGGGUAGAACCACGG	UACCACAGGGUAGAACCACGG	bta-miR-140-3p
4373138	000462	hsa-miR-140	AGUGGUUUUACCCUAUGGUAG	AGUGGUUUUACCCUAUGGUAG	bta-miR-140-5p
4373135	000465	hsa-miR-142-5p	CAUAAAGUAGAAAGCACUAC	CAUAAAGUAGAAAGCACUAC	bta-miR-142-5p
4373134	000466	hsa-miR-143	UGAGAUGAAGCACUGUAGCUCA	UGAGAUGAAGCACUGUAGCUCG	bta-miR-143
4373133	000467	hsa-miR-145	GUCCAGUUUUCCCAGGAAUCCCU	GUCCAGUUUUCCCAGGAAUCCCU	bta-miR-145
4373127	000473	hsa-miR-150	UCUCCCAACCCUUGUACCAGUG	UCUCCCAACCCUUGUACCAGUGu	bta-miR-150
4373179	000596	hsa-miR-151	aCUAGACUGAAGCUCCUUGAGG	CUAGACUGAAGCUCCUUGAGG	bta-miR-151-3p
4408989	002642	hsa-miR-151-5P	UCGAGGAGCUCACAGUCUAGU	UCGAGGAGCUCACAGUCUAGU	bta-miR-151-5p
4395459	002623	hsa-miR-155	UUAAUGCUAAUCGUGAUAGGGGU	UUAAUGCUAAUCGUGAUAGGGGU	bta-miR-155
4373117	000480	hsa-miR-181a	AACAUUCAACGCUGUCGGUGAGU	AACAUUCAACGCUGUCGGUGAGUu	bta-miR-181a
4373116	001098	hsa-miR-181b	AACAUUCAUUGCUGUCGGUGGG	AACAUUCAUUGCUGUCGGUGGGuu	bta-miR-181b
4373112	000486	hsa-miR-186	CAAAGAAUUCUCCUUUUGGGCUu	CAAAGAAUUCUCCUUUUGGGCU	bta-miR-186
4395410	002299	hsa-miR-191	CAACGGAAUCCCAAAAGCAGCUG	CAACGGAAUCCCAAAAGCAGCUG	bta-miR-191
4373108	000491	hsa-miR-192	CUGACCUAUGAAUUGACAGCC	CUGACCUAUGAAUUGACAGCCag	bta-miR-192

4395361	002250	hsa-miR-193a-3p	AACUGGCCUACAAAGUCCCAGU	AACUGGCCUACAAAGUCCCAGU	bta-miR-193a
4395597	002467	mmu-miR-193b	AACUGGCCCACAAAGUCCCGCU	AACUGGCCCACAAAGUCCCGCU	bta-miR-193b
4373104	000495	hsa-miR-196a	UAGGUAGUUUCAUGUUGUUGG	UAGGUAGUUUCAUGUUGUUGG	bta-miR-196a
4395326	002215	hsa-miR-196b	UAGGUAGUUUCCUGUUGUUGGG	UAGGUAGUUUCCUGUUGUUGGG	bta-miR-196b
4373102	000497	hsa-miR-197	UUCACCACCUUCUCCACCCAGC	UUCACCACCUUCUCCACCCAGC	bta-miR-197
4395415	002304	hsa-miR-199a-3p	ACAGUAGUCUGCACAUUGGUUA	ACAGUAGUCUGCACAUUGGUUA	bta-miR-199a-3p
4373272	000498	hsa-miR-199a	CCCAGUGUUCAGACUACCUGUUc	CCCAGUGUUCAGACUACCUGUU	bta-miR-199a-5p
4373100	000500	hsa-miR-199b	CCCAGUGUUUAGACUAUCUGUUC	CCCAGUGUUUAGACUAUCUGUUC	bta-miR-199b
4395417	002306	hsa-miR-214	ACAGCAGGCACAGACAGGCAGU	ACAGCAGGCACAGACAGGCAGU	bta-miR-214
4373077	000524	hsa-miR-221	AGCUACAUUGUCUGCUGGGUUUc	AGCUACAUUGUCUGCUGGGUUU	bta-miR-221
4373076	000525	hsa-miR-222	AGCUACAUCUGGCUACUGGGUCUc	AGCUACAUCUGGCUACUGGGUCU	bta-miR-222
4395388	002277	hsa-miR-320	AAAAGCUGGGUUGAGAGGGCGA	AAAAGCUGGGUUGAGAGGGCGA	bta-miR-320
4378090	001271	hsa-miR-363	AAUUGCACGGUAUCCAUCUGUA	AUUGCACGGUAUCCAUCUGCG	bta-miR-363
4373194	001020	hsa-miR-365	UAAUGCCCCUAAAAAUCCUUAU	UAAUGCCCCUAAAAAUCCUUAU	bta-miR-365
4373028	000563	hsa-miR-374	UUAUAAUACAACCUGAUAAGUG	UUAUAAUACAACCUGAUAAGUG	bta-miR-374a
4381045	001319	mmu-miR-374-5p	AUAUAAUACAACCUGCUAAGUG	AUAUAAUACAACCUGCUAAGUG	bta-miR-374b
4381095	001314	rno-miR-422b	ACUGGACUUGGAGUCAGAAGGC	ACUGGACUUGGAGUCAGAAGGC	bta-miR-378
4381032	001821	hsa-miR-484	UCAGGCUCAGUCCCCUCCCGAU	UCAGGCUCAGUCCCCUCCCGAU	bta-miR-484
4381046	001346	mmu-miR-497	CAGCAGCACACUGUGGUUUGUA	CAGCAGCACACUGUGGUUUGUA	bta-miR-497
4380928	001518	hsa-miR-532	CAUGCCUUGAGUGUAGGACCGU	CAUGCCUUGAGUGUAGGACCGU	bta-miR-532-5p
4395376	002265	hsa-miR-544	AUUCUGCAUUUUUAGCAAGUUC	AUUCUGCAUUUUUAGCAAGUUC	bta-miR-544
4395439	002328	hsa-miR-760	CGGCUCUGGGUCUGUGGGGA	CGGCUCUGGGUCUGUGGGGA	bta-miR-760
4398987	CCS0JFW	bta-un07	AAGAGUUUGUUCGGGUUUCUC	AAGAGUUUGUUCGGGUUUCUC	bta-miR-2284w
4398987	CCRRK9O	bta-un12	CCUCAGUCAGCCUUGUGGAUGU	CCUCAGUCAGCCUUGUGGAUGU	bta-miR-3431
4398987	CCQIM3G	bta-un23	UGAAAAGUUCGUUCGGGUUUUU	UGAAAAGUUCGUUCGGGUUUUU	bta-miR-2284x
4398987	CCO9OW8	bta-un24	UGCGGGAUCUUUAGUUGUGGUG	UGCGGGAUCUUUAGUUGUGGUG	bta-miR-3432
