# Supplementary figures and images for: Characterization of microRNA expression in bovine adipose tissues: a potential regulatory mechanism of subcutaneous adipose tissue development
Source: BMC Mol Biol. 2010 Apr 27;11:29. doi: 10.1186/1471-2199-11-29 (PMC2874793; doi:10.1186/1471-2199-11-29)

## Additional file 2

### The genomic organization of the miR-17-92 family

**miR-17-92**

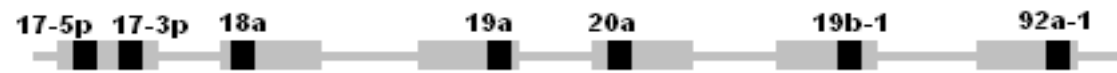

**miR-106a-363**

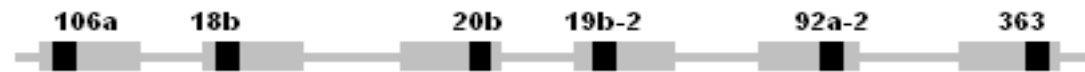

**miR-106b-25**

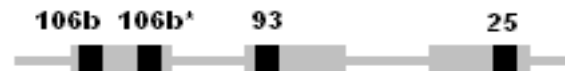

Supplement: Additional file 2 — The genomic organization of the miR-17-92 family. [file 1471-2199-11-29-S2.PDF]
